# Ocular Safety of Intravitreal Engineered Humanized Anti-VEGF Nanobody and Its Efficacy in the Attenuation of Choroidal Neovascularization and Associated Subretinal Fibrosis

**DOI:** 10.3390/biom16060772

**Published:** 2026-05-25

**Authors:** Mir Salar Kazemi, Mozhgan Rezaei Kanavi, Fatemeh Kazemi-Lomedasht, Reza Ahangari Cohan, Golnoosh Mahjoobi, Sare Safi, Sadra Ashrafi, Hamid Ahmadieh, Alireza Shoari, Mahdi Behdani

**Affiliations:** 1 Venom and Biotherapeutics Molecules Laboratory, Biotechnology Research Center, Pasteur Institute of Iran, Tehran 13169-43551, Iran; s_kazemi@pasteur.ac.ir (M.S.K.); fa_kazemi@pasteur.ac.ir (F.K.-L.);; 2 Student Research Committee, Pasteur Institute of Iran, Tehran 13169-43551, Iran; 3 Ocular Tissue Engineering Research Center, Research Institute for Ophthalmology and Vision Science, Shahid Beheshti University of Medical Sciences, Tehran 16666-73111, Iran; rezaeikanavi@sbmu.ac.ir (M.R.K.); sadra.ashrafi@sbmu.ac.ir (S.A.); 4 Nanobiotechnology Department, New Technologies Research Group, Pasteur Institute of Iran, Tehran 13169-43551, Iran; cohan_r@pasteur.ac.ir; 5 Ophthalmic Epidemiology Research Center, Research Institute for Ophthalmology and Vision Science, Shahid Beheshti University of Medical Sciences, Tehran 16666-73111, Iran; safi@sbmu.ac.ir; 6 Ophthalmic Research Center, Research Institute for Ophthalmology and Vision Science, Shahid Beheshti University of Medical Sciences, Tehran 16666-73111, Iran; hahmadieh@sbmu.ac.ir; 7 Department of Cancer Biology, Mayo Clinic, Jacksonville, FL 32224, USA

**Keywords:** choroidal neovascularization, subretinal fibrosis, nanobody, intravitreal injection, angiogenesis, inflammation

## Abstract

Current treatments for choroidal neovascularization (CNV) and its associated subretinal fibrosis (SRF), major causes of vision loss, are limited by the need for frequent intravitreal injections and the emergence of drug resistance. This study evaluated the safety and efficacy of the intravitreal administration of engineered humanized anti-vascular endothelial growth factor Nanobodies, including a wild-type Nanobody (WHNb) and two mutated variants (MHNb136 and MHNb256), in a rat model of laser-induced CNV and associated SRF. Safety was assessed through in vivo electrophysiological and histopathological analyses following intravitreal injection of Nanobodies at doses of 12.5, 25, 50, and 100 µg. Efficacy was evaluated in rat models of laser-induced CNV and SRF using double immunohistochemistry for isolectin B4 and anti-collagen type I on sclerochoroidal flat mounts. Mean CNV and SRF areas in Nanobody-treated groups were compared with those in bevacizumab-treated and sham control groups. None of the Nanobodies showed retinal toxicity in safety assessments. Compared with bevacizumab, MHNb136 and MHNb256 reduced the CNV area by 1.72-fold and 1.8-fold, respectively (both *p* < 0.0001), whereas WHNb showed an effect nearly identical to that of bevacizumab. In addition, 12.5 µg MHNb136 and 100 µg MHNb256 reduced the SRF area by 1.3-fold (*p* = 0.047) and 1.6-fold (*p* = 0.0007), respectively, relative to bevacizumab. For CNV reduction, 12.5 µg MHNb136 was comparable to 25 µg MHNb256; both outperformed bevacizumab. For SRF reduction, 12.5 µg MHNb136 was more effective than bevacizumab and comparable to 100 µg MHNb256. These findings suggest that 12.5 µg MHNb136 may represent a cost-effective bioengineered Nanobody candidate for future clinical studies.

## 1. Introduction

Age-related macular degeneration (AMD), as a growing public health concern, affects millions of aged individuals worldwide, and its burden is projected to markedly increased by 2050 [1]. Choroidal neovascularization (CNV) and associated subretinal fibrosis (SRF), characterized as the progressive degeneration of the neurosensory retina, retinal pigment epithelium, and choriocapillaris complex, are the primary cause of considerable vision loss in neovascular AMD (nAMD) [2,3,4,5,6]. At present, anti-vascular growth factor (VEGF) therapy serves as the cornerstone of managing nAMD; however, SRF, as a manifestation of ongoing or incompletely controlled disease activity, develops in approximately 50% of treated eyes within 2 years of anti-VEGF therapy [7,8]. Additionally, concerns exist regarding the high cost of treatment and the danger of endophthalmitis that results from repeated intravitreal injections [3,9,10,11,12,13,14,15]. Hence, seeking a new therapeutic modality targeting both CNV and associated SRF, while reducing the number of injections, is necessary.

In addition to monoclonal antibodies (mAbs), small antibody fragments have become prevalent binders in treatment and diagnostic applications due to their exceptional selectivity and stability [16,17,18]. Recently, Nanobodies^®^ (VHHs), the smallest intact and natural antigen-binding fragments (~125 residues and ~11–15 kDa), are able to identify specific concealed antigen epitopes and permeate tissues more readily than mAbs [19,20,21]. Moreover, they can withstand heat and chemicals, making them particularly useful in harsh conditions [22,23]. Another advantage of Nanobodies is related to their simple structure and correct folding during bacterial expression, which makes them cost-effective [20,24,25].

Some of the Nanobodies have been approved for cancer immunotherapy and in the treatment of inflammatory diseases [26]. In a previous study, Nanobody 42 (Nb42), as a specific anti-VEGF Nanobody, was shown to potently inhibit human endothelial cell migration and significantly reduced tumor growth in tumor-bearing mice. Given that the efficacy of humanized Nb42 [27] was not investigated in ocular vascular disorders and related fibrosis, the current study was designed to explore the safety and efficacy of the intravitreal injection of bio engineered high affinity humanized anti-VEGF Nanobodies [wild-type Nanobody (WHNb) and mutated Nanobodies (MHNb136 and MHNb256)] in an experimental CNV model in terms of reducing the CNV and SRF areas. MHNb136 and MHNb256, in our previous study [28], were selected using umbrella sampling simulation to improve their binding affinity to VEGF, and confirmed by surface plasmon resonance (SPR) analyses.

## 2. Material and Methods

### 2.1. Study Design

A two-phase experimental study was designed to evaluate the safety of the intravitreal injection of WHNb, MHNb136, and MHNb256 (Phase I), and their inhibitory effect on CNV and related fibrosis in a rat model of laser-induced CNV (Phase II). The WHNb was introduced by our research group previously [20]. The sequence data of mutated humanized Nanobodies, MHNb136 and MHNb256, have been submitted to the GenBank database under accession numbers PV520544 and PV520545, respectively. The research was ethically reviewed and approved by the ethical committee of the Ophthalmic Research Center, Shahid Beheshti University of Medical Sciences (IR.SBMU.ORC.REC.1401.001), and the ethical committee of the Pasteur Institute of Iran (IR.PII.AEC.1401.001). All animal experiments adhered to the Association for Research in Vision and Ophthalmology (ARVO) Statement for the Use of Animals in Ophthalmic and Vision Research and Shahid Beheshti Medical University guidelines on using laboratory animals.

#### 2.1.1. Animal Models Preparation and Grouping

In accordance with the ARRIVE guidelines and the EU Directive 2010/63/EU for animal experiments, 136 Lister Hooded pigmented rats (Razi Vaccine and Serum Production Research Institute, Karaj, Iran) weighing 200–250 g and aged six to eight weeks were recruited for experimental procedures. Twenty-four and 112 rats were used for the safety and efficacy examinations, respectively. The animals were provided with access to water and commercial rodent food (Razi Vaccine and Serum Production Research Institute, Karaj, Iran) ad libitum and were kept in plastic cages with a 12/12-h dark–light cycle. All animals were examined using an indirect ophthalmoscope and a slit lamp biomicroscope prior to the onset of the experiments. The investigation omitted animals with any form of ocular abnormality. For all procedures, the animals were anesthetized with an intramuscular administration of ketamine (Exir Pharmaceutical Co., Borujerd, Iran) (80 mg/kg) and xylazine (Serumwerk Bernburg AG, Bernburg, Germany) (10 mg/kg). At the end of each phase, the animals were euthanized according to the ARVO rules, using the intramuscular administration of ketamine (80 mg/kg) and xylazine (10 mg/kg), followed by intracardiac injection of 2% lidocaine.

#### 2.1.2. Intravitreal Injections

Under general anesthesia, pupillary mydriasis was induced with a topical 1% tropicamide eye drop (Sina Darou, Tehran, Iran). After topical anesthesia with anestocaine 0.5% eye drop (Sina Darou, Tehran, Iran), an expert ophthalmologist, who was masked to the study groups, administered injections under sterile conditions with a Hamilton 5 μL glass needle (Hamilton Company, Reno, NV, USA) located just posterior to the limbus, using a surgical microscope. In each procedure, 1 μL of the material of interest was injected intravitreally, followed by topical administration of a chloramphenicol eye drop 0.5% (Sina Darou, Tehran, Iran).

### 2.2. Phase I: Safety

A dose of 100 μg of each Nanobody was examined intravitreally for safety investigations. Rats were randomized into four groups of 6, which received intravitreal 100 μg WHNb (A), 100 μg MHNb136 (B), 100 μg MHNb256 (C), and formulation buffer (control group, D) in the right eyes. Electroretinography (ERG) and ophthalmic examinations were conducted at the baseline (before intravitreal injection) and on day 28 (after intravitreal injection). Clinical examinations, in terms of occurrences of ocular inflammation, cataract formation, and retinal damage, followed the injections. The animals were euthanized following the last examination; the enucleated globes were processed for routine histopathological examinations, immunohistochemical investigations for glial fibrillary acidic protein (GFAP), and the TUNEL assay.

#### 2.2.1. Electroretinography

Electroretinograms, as described before [28], were acquired at the baseline, immediately prior to injections, and at 4 weeks following the intravitreal injection of wild and mutant Nanobodies. The dark-adapted animals were anesthetized and their pupils were dilated with topical 1% tropicamide. Reference and ground electrodes were subcutaneously implanted behind the ears and the tail, respectively. The Goldring electrodes were positioned on the cornea using hydroxyethyl cellulose gel. The RETI-port/scan 21 electrophysiological diagnostic instruments (Roland Consult Stasche & Finger GmbH, Brandenburg an der Havel, Germany) were used to record the ERG signals. Scotopic responses were recorded for the eyes simultaneously; the photopic responses were recorded consequently after 10 min of light adaptation.

#### 2.2.2. Histopathological and Immunohistochemical Examinations

After the final ERG record, the animals were euthanized and both eyes were promptly enucleated. The globes were then fixed in 10% formalin (Aral Shimi Industries, Gorgan, Iran) for 24 h. To produce thin tissue sections at three different tissue planes (200 μm apart), each globe was bisected axially, processed, and embedded in paraffin blocks. Subsequently, thin (5-μm) tissue sections were subjected to routine hematoxylin and eosin (H and E) staining and immunostaining for GFAP. In immunohistochemistry, the tissue sections were deparaffinized and hydrated via immersion in graded alcohol solutions and xylene. After heat-induced antigen retrieval in sodium citrate buffer, the slides were incubated overnight at 4 °C with a 1:100 dilution of polyclonal rabbit anti-GFAP protein (S0334, Dako/Agilent Technologies, Glostrup, Denmark), followed by secondary antibody, DAB (3,3′-Diaminobenzidine) chromogen staining (Sino Biological, Beijing, China), and counterstaining with hematoxylin.

Light microscopy (BX41, Olympus Corporation, Tokyo, Japan) was employed to assess the presence of retinal hemorrhages, inflammations, necrosis, and atrophy in all H and E-stained and GFAP-immunostained slides by an ophthalmic pathologist (MRK) masked to the study groups. Stained sections were examined under a light microscope; images were captured using a digital camera attached to the microscope (DP12 Microscope Camera, Olympus Corporation, Tokyo, Japan). Immunohistochemical images were quantified using Fiji software (ImageJ2/Fiji distribution; National Institutes of Health, Bethesda, MD, USA; https://imagej.net/software/fiji/ accessed on 12 May 2025 in terms of the area of GFAP-reactive (DAB-stained) retinal Müller cells.

#### 2.2.3. TUNEL Assay

The TUNEL assay was also conducted via the One-step TUNEL In Situ Apoptosis Kit (Green, FITC) (Wuhan Elabscience Biotechnology Co., Ltd., Wuhan, China) on thin tissue sections, based on the manufacturer’s instructions. The positive control was a paraffin-embedded section from a known case of retinoblastoma that underwent TUNEL assay. A paraffin-embedded section from a Nanobody-treated eye that received a label solution instead of the TUNEL reaction mixture was considered as the negative control. A fluorescence microscope (Olympus IX71; Tokyo, Japan) with an excitation wavelength in the range of 450–490 nm, equipped with a digital camera (Olympus U-TV0.63XC; Tokyo, Japan), was used to examine and photograph the TUNEL-stained sections. The number of FITC-stained apoptotic cells at 20× hpf was determined using ImageJ software (https://imagej.net/ij/; National Institutes of Health, Bethesda, MD, USA, accessed on 2 July 2025).

### 2.3. Phase II: Efficacy

After CNV induction with the laser, the rats were randomized into 14 groups of 8, which received 12.5 μg, 25 μg, 50 μg, and 100 μg of intravitreal WHNb, MHNb136, MHNb256, 25 μg of bevacizumab as the conventional treatment, and formulation buffer as the control group, in the right eyes. Although few studies have criticized the use of intravitreal bevacizumab in rodent CNV models [29,30,31], many published papers in the recent decade have used bevacizumab as the conventional treatment in rodents [32,33]. Another rationale for using rat models in the current study was the similar potency of the Nb42 in the inhibition of murine and human VEGF-A [27]. On day 14, all rats were euthanized; the enucleated eyes were subjected to double immunostaining for isolectin B4 and anti-collagen type1 and the preparation of sclerochoroidal flat mounts.

#### 2.3.1. Laser-Induced CNV

This study employed the previously defined protocol to conduct the laser-induced CNV model in rats [28]. Briefly, anesthetized animals with dilated pupils using 1% tropicamide were placed in front of a slit lamp laser (Microlase, Keeler Instruments Inc., USA) delivery system. An 810 nm laser photocoagulation was used to induce six to eight laser-induced lesions around the optic nerve head in the rats’ right eyes (laser power: 150 mW, duration: 100 ms, spot size: 100 μm), displaying as an acute vapor bubble due to Bruch’s membrane rupture.

#### 2.3.2. Double Immunostaining for Isolectin B4 and Anti-Collagen Type1, and Preparation of Sclerochoroidal Flat-Mounts

After fixation in 4% paraformaldehyde for 2 h, posterior sclerochoroidal cups were prepared and transferred to PBS. The dissected eyecups were incubated in blocking buffer (50% fetal calf serum, 20% normal goat serum, and 0.01% Triton X-100 in PBS) at room temperature for 1 h and then incubated with monoclonal anti-collagen type 1 (1:500 in blocking buffer overnight at 4 °C). After washing with PBS, the samples were incubated in the dark with secondary antibody CF^TM^594 anti-mouse IgG1, (Cat # SAB4600326—125 µL, Sigma-Aldrich, St. Louis, MO, USA; 1:500 in PBS containing 20% fetal calf serum, 20% normal goat serum, and 0.01% Triton-X-100) for 2 h at room temperature. After washing with PBS, the samples were incubated with FITC-conjugated *Griffonia simplicifolia* Lectin I (GSL I, isolectin B4; Vector Laboratories, Newark, CA, USA; 1:25 dilution, equivalent to 100 μg/mL in PBS) for 2 h at 37 °C. Finally, each sclerochoroidal cup was spread on a glass slide by making 5 to 6 radial incisions. A coverslip was attached to the tissue using VectaMount AQ (Vector Laboratories, Newark, CA, USA) and the slides were stored in the dark for 2 days at 4 °C. The CNVs in sclerochoroidal flatmount specimens were imaged using a fluorescence microscope equipped with appropriate excitation and output filters and a digital camera (described above). Images were taken at 20× magnification using a blue filter to image vascular segments stained with green fluorescence of isolectin B4 and a red filter to image collagen type 1-positive segments in the choroidal neovascularization. The immunofluorescence staining results were quantified for the measurement of the CNV and SRF areas using ImageJ software. Scale calibration and the establishment of appropriate thresholds were implemented to include valid fluorescence signals in the quantitative area measurement procedure, which was subsequently processed in a standardized manner.

### 2.4. Statistical Analysis

Statistical analyses were performed using IBM SPSS Statistics for Windows, version 25.0. Continuous variables are presented as mean, standard deviation, and range. Prior to group comparisons, data were assessed for distributional assumptions, including normality and homogeneity of variance. When the assumptions for parametric testing were satisfied, one-way analysis of variance (ANOVA) was applied followed by the Newman–Keuls post hoc test for multiple group comparisons. A two-sided *p* value < 0.05 was considered statistically significant.

## 3. Results

### 3.1. Phase I: Safety

No abnormalities were observed in the eyes of the control and Nanobodies groups during the follow-up examinations in Phase I of the study. Slit lamp and funduscopic examinations revealed no obvious cataract or retinal detachment in any of the study groups.

#### 3.1.1. Electroretinography

The comparison of ERG results between the studied groups in Phase I is presented in Table 1. The implicit times and ERG pattern were nearly identical in all groups. The mean difference of scotopic and photopic ERG amplitudes and latencies at the baseline and 4 weeks after intravitreal injection at the specified time points of the right (intervention) and left eyes (control) was not statistically significant (Table 1).

#### 3.1.2. Histopathological Safety of Intravitreal Nanobody Injection

**Figure 1:**
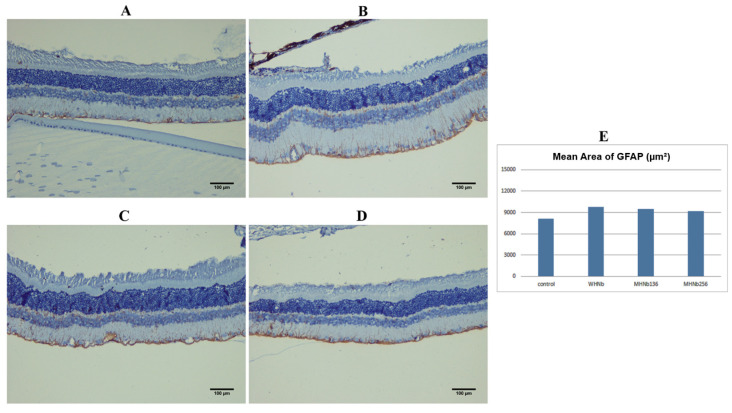
Sample retinal images and Müller cell reactivity of rat retina in the study groups. The integrity of the retinal layers was maintained in the intravitreal injections of sterile formulation buffer in: control (**A**); 100 μg WHNb (**B**); 100 μg MHNb136 (**C**); and 100 μg MHNb256 (**D**) groups. The GFAP-reactivity of retinal Müller cells stained with DAB (brown color) was comparable in all the study groups; (**E**) the average area of GFAP-positive Müller cells in the control (**A**) was not significantly different from that in the WHNb group (**B**), MHNb136 (**C**), and MHNb256 (**D**) groups at a dose of 100 μg.

Overall, the retinal integrity on histopathological examinations was well preserved in the eyes injected with 100 μg WHNb, MHNb136, and MHNb256. Except for two cases with mild intravitreal hemorrhage in the WHNb and one case of mild intravitreal hemorrhage in the MHNb136 group, there was no evidence of necrosis or atrophic retinal changes. The areas of GFAP-positive retinal Müller cells stained with DAB in the eyes injected with 100 μg WHNb (9793.9 ± 3999.3 μm^2^), MHNb136 (9479.8 ± 4253.8 μm^2^), and MHNb256 (9186.8 ± 3669.8 μm^2^) were comparable to the control group (8061.4 ± 3308.3 μm^2^) (*p* = 0.446) (Figure 1).

#### 3.1.3. Intravitreal Nanobody Injection Did Not Induce Retinal Apoptosis

**Figure 2:**
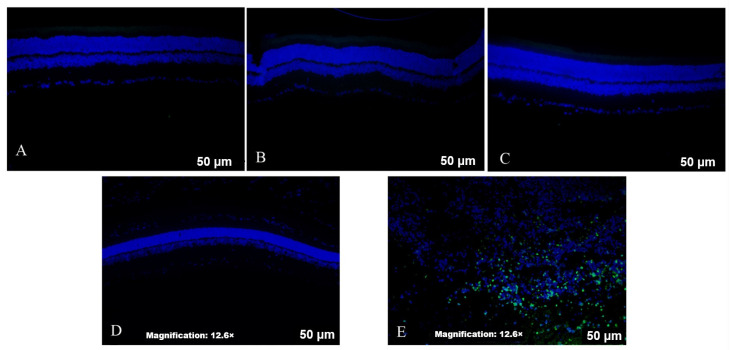
Fluorescent-DAPI composite images of rat retina stained with TUNEL in the study groups. Blue fluorescence indicates DAPI-stained nuclei, and green fluorescence indicates TUNEL-positive apoptotic cells. Images (**A**–**D**) show the absence of apoptotic cells in the retina of rats in the control group (**A**), intravitreal injection of WHNb (**B**), intravitreal injection of MHNb136 (**C**), and intravitreal injection of MHNb256 (**D**) at a dose of 100 μg. Image (**E**) shows a retinoblastoma tissue section as a TUNEL-positive control sample, demonstrating the presence of apoptotic cells.

In the TUNEL evaluations, no apoptotic cells were observed in the retinal tissue of the groups injected intravitreally with the WHNb, MHNb136, and MHNb256 (each at a dose of 100 μg) and the control group (Figure 2).

### 3.2. Phase II: Efficacy

#### 3.2.1. CNV Area

**Figure 3:**
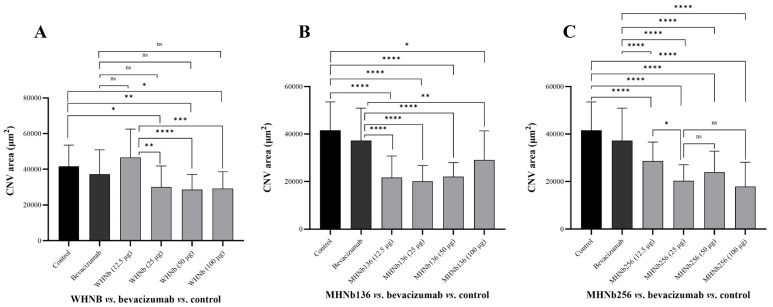
Suppression of CNV and CNV fibrosis following intravitreal administration of Nanobodies in comparison to bevacizumab in a laser-induced CNV rat model: (**A**) WHNB vs. bevacizumab vs. control. The graph shows the CNV area of four doses of intravitreal WHNB vs. bevacizumab and control groups; (**B**) MHNb136 vs. bevacizumab vs. control. The graph shows the CNV area of four doses of intravitreal MHNb136 vs. bevacizumab and control groups; and (**C**) MHNb256 vs. bevacizumab vs. control. The graph shows the CNV area of four doses of intravitreal MHNb256 vs. bevacizumab and control groups. Data are expressed as mean ± SEM. ns, not significant; * *p* < 0.05, ** *p* < 0.01, *** *p* < 0.001, **** *p* < 0.0001.

The mean CNV areas in the study groups, as well as the corresponding representative images, are illustrated in Figure 3. Intravitreal injections of WHNb at doses of 25, 50, and 100 μg significantly attenuated the CNV area in comparison to the control group (*p* = 0.026, *p* = 0.007, and *p* = 0.012, respectively), and as compared to the intravitreal injection of WHNb at the dose of 12.5 μg (*p* = 0.002, *p* = 0.0001, and *p* = 0.001, respectively). Although the mean CNV area in the three groups of intravitreal injection of WHNb at doses of 25, 50, and 100 μg was less than that of the intravitreal injection of bevacizumab, the difference was not statistically significant (*p* = 0.391, *p* = 0.184, and *p* = 0.257, respectively). In addition, the CNV area with intravitreal injection of WHNb at a dose of 12.5 μg did not significantly differ from that in the bevacizumab group (*p* = 0.135), although the former demonstrated a higher mean CNV area. Considering that the mean CNV area did not significantly differ between the doses of 25, 50, and 100 μg, the dose of 25 μg was selected as the minimum effective dose that can decrease the CNV area by 1.23-fold compared to bevacizumab.

Intravitreal injections of MHNb136 at doses of 12.5, 25, 50, and 100 μg significantly attenuated the CNV area in comparison to the control group (*p* = 0.0001, *p* = 0.0001, *p* = 0.0001, and *p* = 0.02, respectively). The mean CNV area of intravitreal injection of MHNb136 at all doses was significantly less than that of the intravitreal injection of bevacizumab (*p* = 0.0001 for doses of 12.5, 25, and 50 μg; *p* = 0.003 for dose of 100 μg). Given that the mean CNV area was not significantly different between all doses, the dose of 12.5 μg was selected as the minimum effective dose that can decrease the CNV area by 1.72-fold compared to bevacizumab.

Intravitreal injections of MHNb256 at doses of 12.5, 25, 50, and 100 μg significantly attenuated the CNV area in comparison to the control group (*p* = 0.0001 in all groups). Similarly, the mean CNV area in all groups of MHNb256 was significantly less than that of the intravitreal injection of bevacizumab (*p* = 0.0001 in all groups). Considering that the effectiveness of a dose of 25 μg was comparable to doses of 50 and 100 μg (*p* = 0.907 and *p* = 0.958, respectively) and was higher than a dose of 12.5 μg (*p* = 0.016), the dose of 25 μg was selected as the minimum effective dose that can decrease the CNV area by 1.8-fold compared to bevacizumab. The intravitreal dose of MHNb136 at 12.5 μg was as effective as MHNb256 at 25 μg in the attenuation of CNV areas (*p* = 0.485). Because several doses showed broadly similar efficacy, the minimum effective dose for a significant decrease in the CNV area as the prespecified exploratory efficacy criterion was considered in this preclinical study.

#### 3.2.2. CNV Fibrosis Area

**Figure 4:**
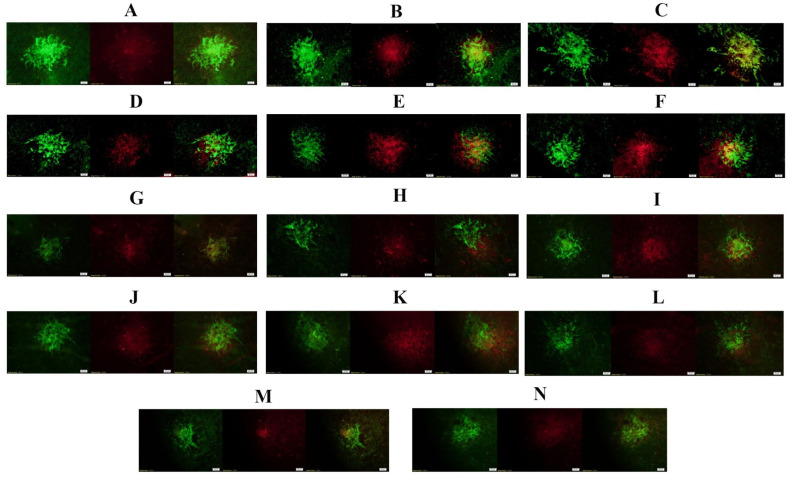
The effects of WHNb, MHNb136, and MHNb256 on CNV and SRF in laser-induced CNV rats. Columns from left to right: isolectin B4 (green fluorescence) to label laser-induced CNVs, anticollagen (red fluorescence) to label CNV-associated fibrosis, and merged images of isolectin and anticollagen type 1 staining. Rows from top to bottom: (**A**) control (sterile formulation buffer), (**B**) intravitreal bevacizumab (IVB); and various concentrations of WHNb with (**C**) 12.5 μg, (**D**) 25 μg, (**E**) 50 μg, and (**F**) 100 μg; various concentrations of MHNb136 with (**G**) 12.5 μg, (**H**) 25 μg, (**I**) 50 μg, and (**J**) 100 μg; and various concentrations of MHNb256 with (**K**) 12.5 μg, (**L**) 25 μg, (**M**) 50 μg, and (**N**) 100 μg, respectively. The scale bar in each image is 50 μm.

**Figure 5:**
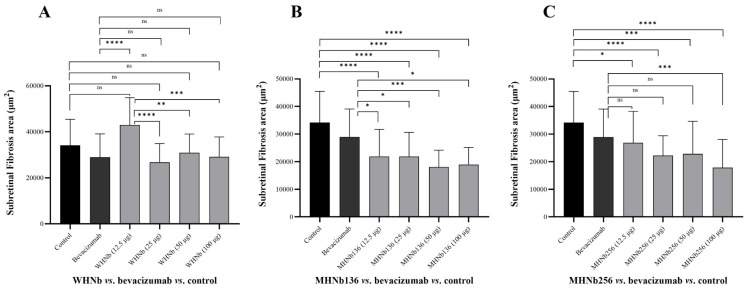
Suppression of CNV fibrosis following intravitreal administration of Nanobodies in comparison to bevacizumab in a laser-induced CNV rat model: (**A**) WHNB vs. bevacizumab vs. control. The graph shows the CNV fibrosis area of four doses of intravitreal WHNB vs. bevacizumab and control groups; (**B**) MHNb136 vs. bevacizumab vs. control. The graph shows the CNV fibrosis area of four doses of intravitreal MHNb136 vs. bevacizumab and control groups; and (**C**) MHNb256 vs. bevacizumab vs. control. The graph shows the CNV fibrosis area of four doses of intravitreal MHNb256 vs. bevacizumab and control groups. Data are expressed as mean ± SEM. ns, not significant; * *p* < 0.05, ** *p* < 0.01, *** *p* < 0.001, **** *p* < 0.0001.

The mean areas of CNV fibrosis in study groups and their corresponding representative images are shown in Figure 4 and Figure 5. The mean areas of CNV fibrosis in all WHNb groups were not significantly different from those of the controls (*p* = 0.05, *p* = 0.143, *p* = 0.890, and *p* = 0.548 for 12.5, 25, 50, and 100 μg, respectively). However, the mean areas of CNV fibrosis at the dose of 12.5 μg (*p* = 0.0001) were significantly higher than those in the bevacizumab group. The intravitreal injections of other doses of WHNb demonstrated similar effects on the attenuation of CNV fibrosis compared to the bevacizumab group (*p* = 0.979, *p* = 0.986, and *p* = 0.999 for 25, 50, and 100 μg, respectively) and a significant effectivity in comparison to WHNb at the dose of 12.5 μg (*p* = 0.0001, *p* = 0.006, and *p* = 0.001, respectively). Therefore, the dose of 25 μg was the minimum effective dose for attenuating CNV fibrosis by 1.08-fold compared with bevacizumab.

The mean area of CNV fibrosis was significantly reduced in the intravitreal injection of MHNb136 at all doses in comparison to the controls (*p* = 0.0001 in all groups) and the intravitreal bevacizumab group (*p* = 0.047, *p* = 0.015, *p* = 0.001, and *p* = 0.014 for 12.5, 25, 50, and 100 μg, respectively). Given that the mean areas of CNV fibrosis did not significantly differ between all MHNb136 doses, the dose of 12.5 μg was selected as the minimum effective dose that can decrease the CNV fibrosis area by 1.32-fold compared to bevacizumab.

Intravitreal MHNb256 at all doses of 12.5, 25, 50, and 100 μg significantly reduced the mean area of CNV fibrosis in comparison to the control (*p* = 0.017, *p* = 0.0001, *p* = 0.0003, and *p* = 0.0001, respectively). Although e intravitreal MHNb256 at the dose of 100 μg was superior to bevacizumab in reducing CNV fibrosis (*p* = 0.0007), other doses did not significantly differ from the bevacizumab group (*p* = 0.925, *p* = 0.06, and *p* = 0.144 for 12.5, 25, and 50 μg, respectively). MHNb256 at the dose of 100 μg was the effective dose in attenuating the CNV fibrosis by 1.6-fold compared to bevacizumab. However, the inhibitory effect of 12.5 μg MHNb136 on CNV fibrosis was similar to all doses of MHNb256 (*p* = 0.209, *p* = 0.580, *p* = 0.776, and *p* = 0.241 for doses of 12.5, 25, 50, and 100 μg, respectively).

The minimum effective dose for a significant decrease in the SRF area, as another prespecified exploratory efficacy criterion in this study, was considered. Given the superiority of MHNb136 to MHNb256 on SPR analyses, the similar effectiveness of MHNb136 of 12.5 μg and MHNb256 of 25 μg in the attenuation of the CNV area, the superiority of 12.5 μg MHNb136 to bevacizumab in reducing the CNV fibrosis area, and the cost-effectiveness of the dose of 12.5 μg, the MHNb136 dose of 12.5 μg was selected for further clinical studies.

## 4. Discussion

Our investigations demonstrated the in vivo safety of WHNb, MHNb136, and MHNb256 when administered intravitreally. The safety data in this study were encouraging, using ERG, histology, GFAP staining, and TUNEL assay, and, similar to prior studies [34,35], included an acceptable follow-up period. An intravitreal dose of MHNb136 of 12.5 μg was as effective as a dose of MHNb256 of 25 μg in the attenuation of both CNV and CNV-related fibrotic areas and was superior to the WHNb and bevacizumab groups. Although a variety of anti-VEGF antibodies have been using for the treatment of nAMD [7], to the best of our knowledge, our study is the first in vivo preclinical investigation that explored the effectiveness of anti-VEGF humanized Nanobodies on reducing CNVs and CNV-related subretinal fibrosis. These Nanobodies can be promising alternatives for conventional anti-VEGF therapies in nAMD due to their smaller size, enhanced tissue penetration, heat resistance, and simpler production methods, which offer potential improvements in both efficacy and cost-effectiveness [36,37].

Currently, anti-VEGF therapy is the primary treatment option for active CNV; however, CNV-associated SRF may develop in approximately half of cases during anti-VEGF therapy [38]. The SRF, similar to CNV, has been recognized as a significant cause of blindness. More clinical ophthalmologists are realizing that the inhibition of SRF may be as significant as anti-VEGF treatments [38]. There have been multiple preclinical studies on the efficacy of new and alternative treatments for the mitigation of CNV fibrosis [39]. The investigated Nanobodies in our study should be added to the list of new treatments for attenuating CNV-related fibrosis for further clinical settings, especially in CNV cases resistant to conventional therapies.

Because of the advantages of Nanobodies over conventional therapeutic antibodies, the use of Nanobodies in various diseases has been increasing. For instance, Cablivi^®^, or caplacizumab, was the first biopharmaceutical Nanobody available in the United States and Europe that is used for the treatment of acquired thrombocytopenic purpura [40,41]. Envafolimab (anti-programmed death ligand 1 Nanobody) and Ozoralizumab (trivalent anti-tumor necrosis factor alpha Nanobody) are other examples of Nanobodies approved for cancer immunotherapy and in the treatment of inflammatory diseases, respectively [42,43]. In the current study, the effects of intravitreal MHNb136 and MHNb256, as humanized and affinity-matured anti-VEGF Nanobodies, in reducing CNV and related SRF were superior to conventional treatment. This underscores the potential use of intravitreal Nanobodies, especially MHNb136, for future targeted therapies, not only in nAMD, but also in other angiogenesis-related ocular diseases such as diabetic retinopathies or retinopathy of prematurity.

Given the efficacy of 12.5 μg MHNb136 on the reduction in CNV and CNV-related SRF areas compared to conventional treatment, WHNb, and MHNb256 in rat models, and considering the cost-effectiveness of the dose of 12.5 μg MHNb136 compared to other Nanobodies, it can be a promising candidate for future clinical studies. In comparison to other anti-VEGF medications, such as bevacizumab (150 kDa), aflibercept (115 kDa), ranibizumab (48 kDa), and brolucizumab (26 kDa), the Nanobody (15 kDa) has a smaller molecular weight, and theoretically can have a higher concentration of VEGF inhibitors in ocular fluids. This may lead to faster clearance times of the Nanobody; however, due to its higher equivalent concentrations compared to other anti-VEGF medications, it has a higher number of VEGF inhibitory bindings, and, subsequently, a longer duration of impact [41]. Another advantage of Nanobodies, as demonstrated by Muyldermans [19], is their stability and lack of immunogenicity, which make them a superior treatment to other medications in various VEGF signaling-related disorders. The Nanobodies, due to their small sizes, can penetrate tissues more easily and reach targets that are unreachable by larger-sized molecules. Given that inflammations and subsequent macrophage activation are critically involved in anti-VEGF resistant cases [44], the lack of immunogenicity is another advantage of Nanobodies over other anti-VEGF medications, making them promising in CNV cases resistant to conventional intravitreal treatments. However, a lack of knowledge on the intraocular half-life of the selected dose of MHNb136 and its pharmacokinetics is the main drawback of our preclinical study that should be resolved in future studies. Moreover, the laser-induced CNV rat model is suitable for proof-of-concept efficacy testing and may not establish clinical readiness.

## 5. Conclusions

The current study demonstrates the ocular safety and preclinical efficacy of engineered humanized affinity-matured anti-VEGF Nanobodies following intravitreal administration. Safety assessments, including electrophysiological, histopathological, GFAP immunostaining, and TUNEL analyses, showed no evidence of retinal toxicity, structural damage, gliotic activation, or apoptosis after the intravitreal injection of WHNb, MHNb136, or MHNb256, even at the highest tested dose. In the laser-induced CNV rat model, the affinity-matured variants, particularly MHNb136 and MHNb256, produced stronger anti-angiogenic and anti-fibrotic effects than WHNb and bevacizumab. Notably, 12.5 μg MHNb136 significantly reduced both CNV and CNV-associated SRF areas and showed efficacy comparable to 25 μg MHNb256 for CNV suppression, while also outperforming bevacizumab in reducing fibrosis. Given its favorable safety profile, strong efficacy at the lowest tested dose, and potential cost-effectiveness, intravitreal MHNb136 at 12.5 μg represents a promising candidate for further pharmacokinetic, dose-optimization, and translational studies. These findings support continued development of humanized anti-VEGF Nanobodies as next-generation biologic therapeutics for CNV, CNV-associated fibrosis, and potentially other angiogenesis-driven ocular diseases.

## Figures and Tables

**Table 1:** Comparison of ERG results between the studied groups in Phase I.

	WHNb (Mean ± SD)			MHNb136 (Mean ± SD)			MHNb256 (Mean ± SD)		
	Intervention	Control	*p*-Value	Intervention	Control	*p*-Value	Intervention	Control	*p*-Value
Scotopic									
0.01—Latency–B-wave	1.63 ± 16.15	−1.65 ± 8.99	0.3125	6.80 ± 15.87	4.42 ± 7.59	1.000	−18.23 ± 15.87	−18.40 ± 13.51	1.000
0.01—Amplitude–B-wave	−27.32 ± 61.74	−24.23 ± 106.21	1.0000	−22.90 ± 14.14	−9.07 ± 16.25	0.125	−96.38 ± 49.12	−54.95 ± 48.89	0.250
1—Latency–A-wave	1.92 ± 3.95	−0.40 ± 1.99	0.3125	1.45 ± 6.49	1.92 ± 4.42	0.875	−0.03 ± 4.53	0.28 ± 2.00	0.875
1—Latency–B-wave	2.17 ± 5.07	−1.90 ± 7.34	0.8438	−4.82 ± 9.29	−6.92 ± 8.24	0.625	3.95 ± 3.63	0.75 ± 4.79	0.625
1—Amplitude–A-wave	−20.38 ± 51.18	7.37 ± 68.69	1.0000	−7.65 ± 33.50	2.70 ± 25.85	0.875	−21.70 ± 27.88	6.80 ± 28.46	0.375
1—Amplitude–B-wave	−42.40 ± 88.93	−11.43 ± 144.33	1.0000	−13.10 ± 57.98	−2.65 ± 52.93	0.625	−69.30 ± 39.11	−8.90 ± 37.05	0.125
1—b/a	0.35 ± 1.03	−0.18 ± 0.63	0.4375	1.10 ± 3.55	0.22 ± 1.56	1.000	−0.65 ± 0.77	−1.00 ± 2.18	1.000
10—Latency–A-wave	1.83 ± 6.33	−0.38 ± 2.79	0.5625	−0.05 ± 6.20	−1.98 ± 2.10	0.625	3.08 ± 7.72	2.70 ± 7.99	1.000
10—Latency–B-wave	−2.70 ± 9.77	0.53 ± 2.43	0.4185	−8.50 ± 11.53	−6.52 ± 8.09	0.875	−4.90 ± 10.02	−9.00 ± 3.64	0.375
10—Amplitude–A-wave	−18.45 ± 49.84	−3.72 ± 87.40	1.0000	−33.50 ± 74.07	−3.53 ± 14.60	0.375	−41.00 ± 50.28	0.57 ± 34.07	0.250
10—Amplitude–B-wave	−37.83 ± 97.10	−21.57 ± 128.39	1.0000	−23.58 ± 17.74	30.68 ± 40.63	0.125	−118.25 ± 42.62	−42.98 ± 29.30	0.125
3—Latency–P2	3.75 ± 3.41	2.68 ± 4.39	0.6875	1.42 ± 3.94	−0.85 ± 5.80	0.375	0.52 ± 1.37	2.17 ± 3.77	0.625
3—Amplitude–OS2	−6.42 ± 47.93	3.30 ± 35.77	0.8438	6.25 ± 7.87	12.62 ± 9.18	1.000	−2.93 ± 21.85	5.50 ± 28.95	0.625
**Photopic**									
3—Latency–A-wave	1.87 ± 11.90	−2.47 ± 8.68	0.5625	5.40 ± 8.86	1.75 ± 9.54	0.250	−6.68 ± 7.59	−5.45 ± 7.54	0.875
3—Latency–B-wave	−0.28 ± 2.91	−7.47 ± 14.50	0.4375	−0.72 ± 5.73	−1.85 ± 5.18	0.875	0.10 ± 6.77	10.00 ± 10.91	0.250
3—Amplitude–A-wave	2.68 ± 11.37	−2.55 ± 10.91	0.6875	−18.53 ± 53.23	5.30 ± 17.59	0.500	12.25 ± 51.22	6.10 ± 43.97	0.625
3—Amplitude–B-wave	−7.20 ± 39.13	−11.08 ± 35.81	1.0000	−28.30 ± 38.15	−15.32 ± 38.84	0.375	−25.52 ± 43.68	2.75 ± 30.44	0.125
30—Latency–P1	2.58 ± 19.19	3.75 ± 13.25	0.8438	−3.33 ± 8.17	7.25 ± 12.90	0.250	0.80 ± 15.76	6.88 ± 11.89	0.375
30—Latency–N1-P1	−0.05 ± 4.58	−0.28 ± 5.98	0.9163	2.12 ± 3.66	6.88 ± 6.64	0.625	3.08 ± 3.50	2.65 ± 6.42	1.000

## Data Availability

All data generated or analyzed during this study are included in this published article. Further inquiries can be directed to the corresponding author.
